# Exploring the role of acylated ghrelin and gut microbiome in delineating cognitive health in the elderly

**DOI:** 10.18632/aging.206200

**Published:** 2025-02-07

**Authors:** Sudeshna Rout, Rishikesh Dash, Varsha Satish, Giriprasad Venugopal, Bodepudi Narasimha Rao, Debapriya Bandhyopadhyay, Sanjeev Kumar Bhoi, Balamurugan Ramadass

**Affiliations:** 1 Department of Biochemistry, All India Institute of Medical Sciences Bhubaneswar, Odisha 751019, India; 2 Centre of Excellence for Clinical Microbiome and Research (CCMR), All India Institute of Medical Sciences Bhubaneswar, Odisha 751019, India; 3 Department of Physiology, All India Institute of Medical Sciences Bhubaneswar, Odisha 751019, India; 4 Department of Neurology, All India Institute of Medical Sciences Bhubaneswar, Odisha 751019, India; 5 Adelaide Medical School, Faculty of Health and Medical Sciences, The University of Adelaide, Adelaide, SA 5000, Australia

**Keywords:** gut microbiome, gut-brain axis, ghrelin, AG/UAG ratio, neural network model, dementia

## Abstract

Introduction: With increased life expectancy, there is an increase in aging population and prevalence of dementia. Ghrelin is a key regulator of spatial memory and cognition. The gut microbiome may affect the circulating levels of unacylated ghrelin (UAG) and acylated ghrelin (AG). Thus, we explore the potential association of the gut microbiome, AG, and cognitive health in the aging dementia patient.

Methods: 40 dementia patients and 40 controls were recruited. Fecal Microbiome analysis using 16S rRNA sequencing was performed on 18 samples. A mixed-method approach was employed for robust interpretation.

Results: Dementia patients had an increased serum AG and AG/UAG ratio. With the increase in AG among dementia subjects, a significant decrease in species richness was observed. *Bifidobacterium longum, Eubacterium biforme, Fecalibacterium prausnitzii, Lactobacillus ruminis*, and *Prevotella copri* contributed to substantial differences in beta-diversity. *Blautia obeum* was associated with Mini-Mental State Examination (MMSE), and *Fecalibacterium prausnitzii* was associated with Montreal Cognitive Assessment (MoCA) Scale.

Discussion: This pilot study indicates a complex interaction between AG, gut microbiome, and cognitive scores. Increased AG corresponds to both dementia and gut dysbiosis, intricately interconnecting the gut-brain axis. The circulating AG and associated gut microbiome might be a putative biomarker for dementia.

## INTRODUCTION

Dementia is primarily a clinical condition affecting the aging population and is typically chronic and progressive. The preclinical stage may be characterized by mild cognitive impairment, frequently accompanied by behavioural abnormalities. Most functioning capacities are severely compromised in the later stages of dementia, leading to considerable decline and eventually complete dependency. Neuropathological abnormalities can begin as early as 20 years before clinical manifestation [1]. Both external and internal factors play a role in developing the disease process. Microbes settled in the gut serve as a connecting link between the external and internal environment, and they act through humoral and vagal pathways to establish a gut-brain axis. Microbes influence the gut-brain axis through secretion of various metabolites like short chain fatty acids (SCFA) and other biologically active molecules like gamma amino butyric acid (GABA), serotonin, dopamine [2]. Notably, ghrelin, a hormone secreted by enteroendocrine cells of stomach is decreased with increase in SCFA like acetate, butyrate and propionate [3]. The gut hormone ghrelin plays a vital role in this gut-brain axis. UAG represents ∼80–90% of circulating ghrelin. UAG undergoes a unique post-translational modification in which the peptide gets acylated, primarily by octanoic acid, on a serine residue over the third amino acid to produce AG. Studies linked to this hormone found no significant difference in total ghrelin levels in dementia. Instead, there was an increase in AG and a subsequent decrease in UAG levels in the cases [4]. In contrast, another study reported a notable reduction in plasma AG/UAG ratio in the Parkinson’s disease dementia (PDD) group compared to the PD group with intact cognition and controls, which contradicts the earlier findings [5]. Studies also provide evidence that pro-inflammatory dysbiosis promotes the development of Parkinson’s disease (PD) by interfering with intestinal permeability [6]. Reduced microbial diversity in the gut, with a notable shift toward pro-inflammatory taxa is also frequently observed in dementia, and Alzheimer’s patients [7]. Gut microbiota and their metabolites can alter the signaling of the ghrelin receptor [8–10]. However, it is unclear whether gut dysbiosis and ghrelin interact in aging dementia subjects. In this study, we aim to investigate the interactions of microbial composition and diversity and the gut hormone ghrelin in aging dementia patients with MMSE scores between 15 and 25 to further delineate their cognitive health.

## RESULTS

### Serum acylated ghrelin significantly increased in dementia

**Figure 1:**
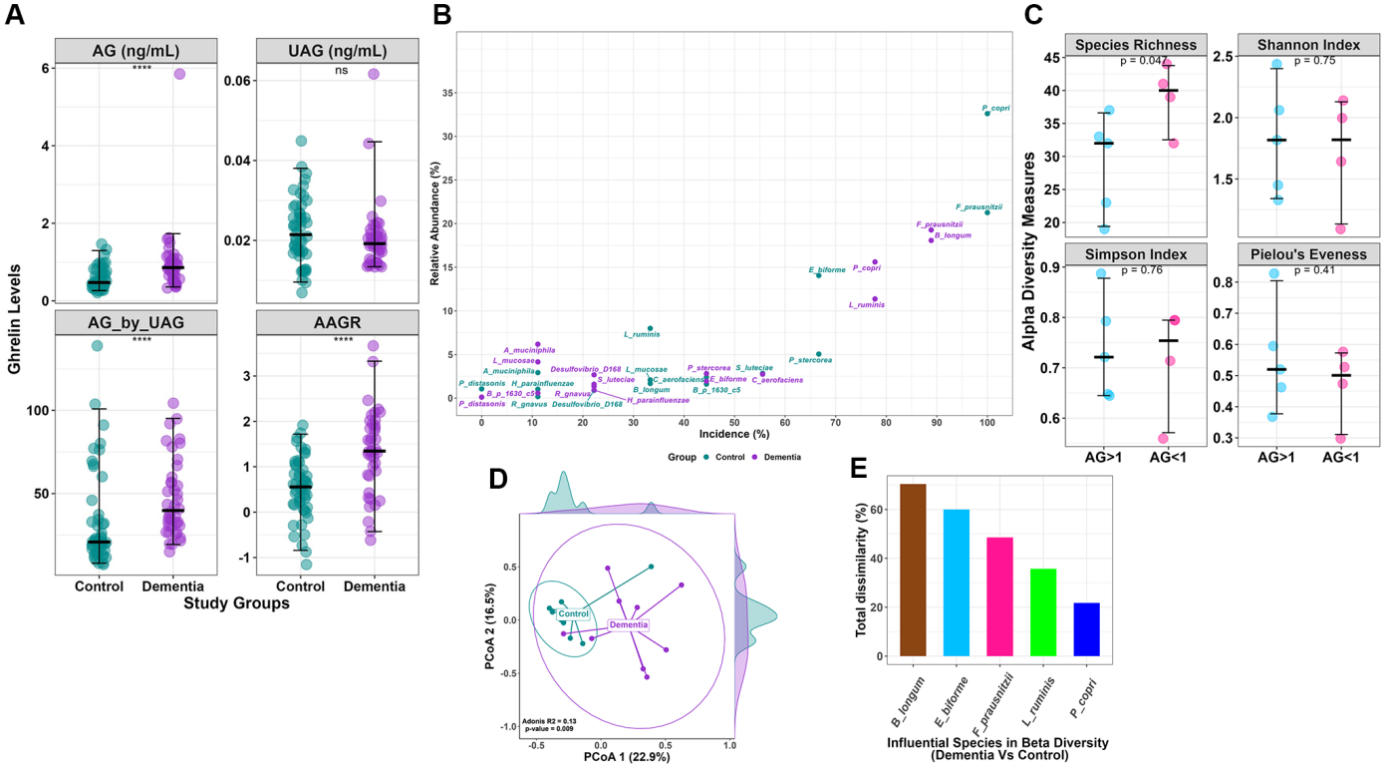
(**A**) Clinical data comparison (AG, UAG, AG/UAG, AAGR) between dementia and controls. (**B**) Relative abundance vs. incidence for the top 15 abundant species. Incidence refers to the number of patients in which a species is observed when its abundance is >1%. (**C**) Alpha diversity indices (Species Richness, Shannon Index, Simpson Index, and Pielou’s Evenness index) with statistical comparison between AG >1 and AG <1 within the dementia group. AG, Acylated Ghrelin. (**D**) Beta diversity using Bray-Curtis dissimilarity is plotted on the principal coordinate axis (PCoA), with density labelled on each axis to represent the variation between sample points for each group. (**E**) Influential species with Bray-Curtis dissimilarity (%) in beta diversity differences between the dementia and control groups.

Dementia clinical assessment scorings like MMSE, MoCA, GADL, and HADS were used to diagnose dementia from the study participants, and dementia patient scores were found to differ significantly from the controls (Table 1). AG (ng/mL) was higher among the cases than in the controls (0.97 ± 0.8 vs. 0.58 ± 0.28, *p* = 0.007), whereas the levels of UAG (ng/mL) were insignificant. The ratio of AG/UAG in dementia subjects was significantly higher than the controls (47.0 ± 23.2 vs. 32.9 ± 28.3, *p* = 0.019). When adjusted for age, the age-adjusted ghrelin ratio (AAGR) was also significantly different between cases and controls (1.33 ± 0.93 vs. 0.53 ± 0.68) (Figure 1A). To exclude the metabolic alteration of ghrelin, we estimated insulin, which was found to be insignificant.

**Table 1:** Demographic and clinical parameter comparison in cases and controls.

**Parameters**		**Overall** **(*n* = 80)**	**Controls** **(*n* = 40)**	**Cases** **(*n* = 40)**	***p*-value^#^**
**Categorical *n* (%)**				
Sex	Male	53 (66.25)	24 (72.5)	29 (72.5)	0.344^a^
	Female	27 (33.75)	16 (27.5)	11 (27.5)	
Education	None	5 (6.25)	2 (7.5)	3 (7.5)	0.964^a^
	Till Matriculation	48 (60)	24 (60)	24 (60)	
	Higher Secondary	13 (16.25)	7 (15)	6 (15)	
	Graduation	14 (17.5)	7 (17.5)	7 (17.5)	
Alcohol	No	73 (91.25)	37 (90)	36 (90)	1^a^
	Yes	7 (8.75)	3 (10)	4 (10)	
Smoking	No	76 (95)	39 (92.5)	37 (92.5)	0.615^a^
	Yes	4 (5)	1 (7.5)	3 (7.5)	
**Continuous mean (range)**				
Age (in years)		67.7 (60–85)	69.3 (60–85)	66.2 (60–81)	<0.001^b^
MMSE Score		26 (15–30)	29 (26–30)	19 (15–25)	<0.001^b^
MoCA Score		26 (11.5–30)	28 (26–30)	21 (11.5–25)	<0.001^b^
GADL Score		23 (8.5–27)	26 (25.5–27)	13.05 (8.5–20)	<0.001^b^
HADS - A Score		5 (0–12)	2 (0–4)	9.5 (7–12)	<0.001^b^
HADS - D Score		5 (0–11.5)	2 (0–3)	9 (8–11.5)	<0.001^b^
AG (ng/mL)		0.75 (0.21–5.85)	0.58 (0.21–1.47)	0.97 (0.33–5.85)	<0.001^b^
UAG (ng/mL)		0.02 (0.007–0.062)	0.022 (0.007–0.045)	0.021 (0.013–0.062)	0.2^b^
AG/UAG ratio		39 (7–139)	33 (7–139)	47 (15–104)	<0.001^b^
AAGR		0.88 (−1.15–3.67)	0.53 (−1.15–1.92)	1.33 (−0.62–3.67)	<0.001^b^
Insulin (µIU/mL)		9.78 (0.98–29.98)	9.56 (1.31–29.98)	10 (0.98–26.61)	0.92^b^

^#^Significance based on *p*-value, *p* < 0.05 is significant. ^a^Chi-Square test. ^b^Student *t*-test. Abbreviations: AG: Acylated Ghrelin; UAG: Unacylated Ghrelin; AG/UAG: Acylated Ghrelin to Unacylated Ghrelin ratio; AAGR: Age Adjusted Ghrelin Ratio; *n*: sample size.

### Bacterial taxonomy identification using 16S rRNA sequencing

Microbiome analysis was performed on nine participants from both study groups. On average, 295,684 reads per sample were obtained using the Illumina MiSeq. At the Phylum level, 24 OTUs were identified, with Firmicutes, Bacteroidetes, Actinobacteria, Proteobacteria, and Verrucomicrobia ranking as the top 5 phyla across all groups. Taxonomic annotation revealed the presence of 93 OTUs at the species level. Following data filtration, 53 OTUs accounting for 99% of total counts at species level were retained and used for all microbiome analyses.

### Variation in microbiota abundance and incidence

The core microbiota of the top 15 species shows distinctions in both abundance and incidence between the control and dementia groups (Figure 1B). Certain microbes, such as *Prevotella copri,* exhibit 16% abundance and are present in over 78% of the dementia group, while in the control group, they are 33% abundant and are present in all (100%). Similarly, *Bifidobacterium longum* displays 2% and 18% abundance in the control and dementia groups, with distinctions in incidence at 33% and 89%, respectively. Meanwhile, *Eubacterium biforme* shows 14% and 2% abundance in the control and dementia groups, with incidence rates of 67% and 44%, respectively. *Lactobacillus ruminis* has 8% and 11% abundance in the control and dementia groups, with incidence rates of 33% and 78%, respectively.

### Distinct microbial diversity identified in dementia

Alpha diversity indices did not show any differences between the control and dementia groups (Supplementary Figure 1A); however, comparing within dementia subgroup based on AG levels (AG >1 ng/ml and AG <1 ng/ml), species richness (28.8 ± 7.5 vs. 39 ± 5.1, *p* = 0.04) (Figure 1C) was found to be significantly different. In contrast, beta diversity showed a significant difference between control and dementia groups (*p* = 0.009) (Figure 1D). A SIMPER test identifies five species that are determinants of beta diversity differences between the control and dementia groups based on the Bray-Curtis dissimilarity index (Figure 1E); they are *Bifidobacterium longum* (70%), *Eubacterium biforme* (60%), *Fecalibacterium prausnitzii* (49%), *Lactobacillus ruminis* (36%), and *Prevotella copri* (22%). The log-transformed F/B ratio showed no differences between the control and dementia groups (Supplementary Figure 1B). No differences were observed in beta diversity in dementia subgroups based on AG.

### Species-specific community interaction identified using co-occurrence network

**Figure 2:**
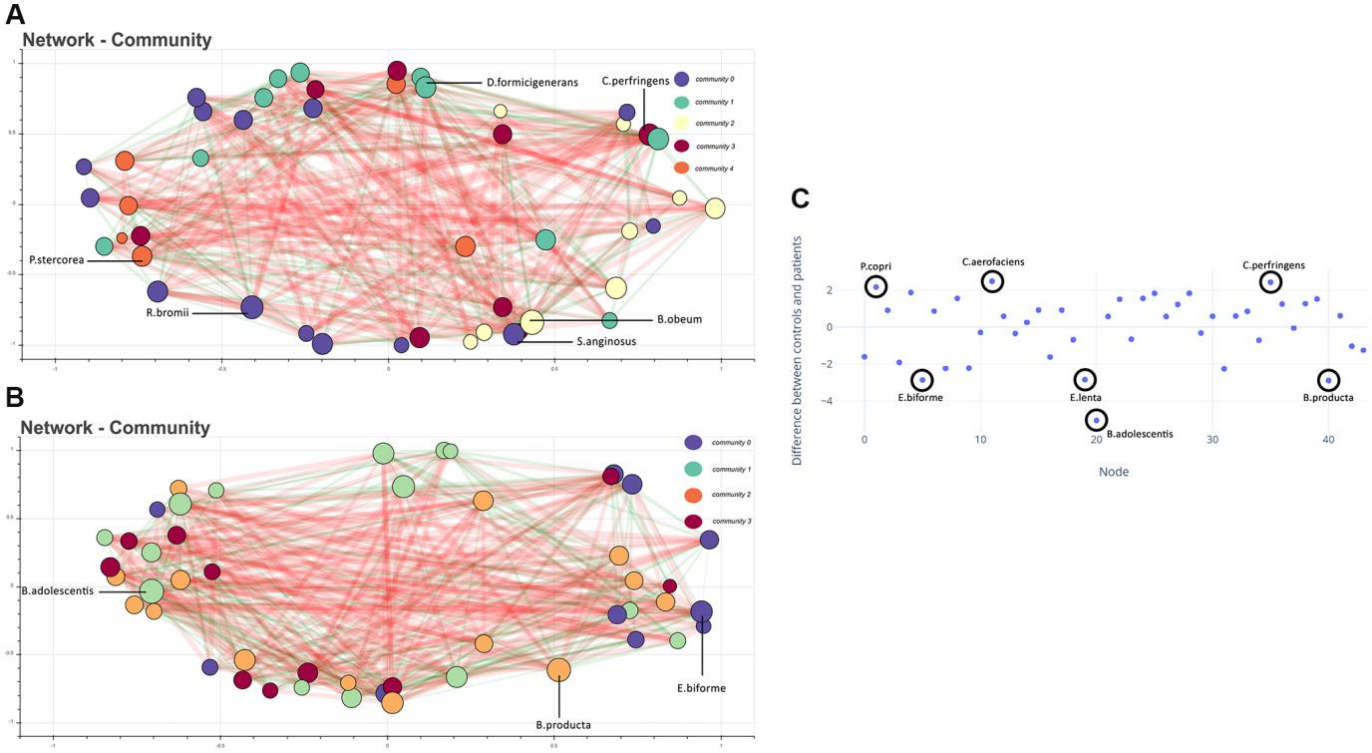
(**A**) Co-occurrence network showing bacteria (with total abundance >10) in the control group. Each node represents a bacteria, and the color represents the community it belongs to. The size of the node is proportional to the degree of the node. Edges are coloured green and red, indicating positive and negative co-occurrence, respectively. The thickness of the edge indicates the strength of the association. Representative high-degree bacteria from each community are labeled. (**B**) Co-occurrence network of microbes in the dementia group. (**C**) Difference in normalized degree centrality for each node between control and dementia networks. Positive values indicate a higher degree of centrality in the control network. Highlighted nodes correspond to outlier species.

Species co-occurrence network analysis shows representative high-degree species in the control network. Communities of microbes were formed within each network according to the Louvain method. In each community, the bacteria with the highest degree of centrality were labelled in the network as representative of that community if it were amongst the top 20 species when arranged in descending order. In controls, *Dorea formicigenerans, Clostridium perfringens, Blautia obeum*, *Streptococcus anginosus, Ruminococcus bromii,* and* Prevotella stercorea* were representative (Figure 2A). Whereas in the dementia group, *Bifidobacterium adolescentis, Blautia producta,* and* Eubacterium biforme* (Figure 2B) were found to be representative. The top 20 species in the dementia group did not include species representing community 3, when analyzed according to the degree of centrality. The disparate importance of certain bacteria in healthy and dementia networks was analysed using a metric of species influence referred to as the degree of centrality. We used the degree of centrality as an indicator of keystone species, with species having a higher degree of centrality in one network than the other, implying the increased influence of that species in the particular group. The degree of centrality was normalized, and their difference in dementia and controls was calculated to identify outliers that referred to bacteria of vastly differing importance in the two networks. We identified that *Prevotella copri, Collinsella aerofaciens,* and *Clostridium perfringens* had enhanced centrality in the control network. *Eubacterium biforme, Eggerthella lenta, Blautia producta,* and *Bifidobacterium adolescentis* were enhanced in the dementia network (Figure 2C).

### MoCA and 14 OTUs contribute to the neural network model decision

**Figure 3:**
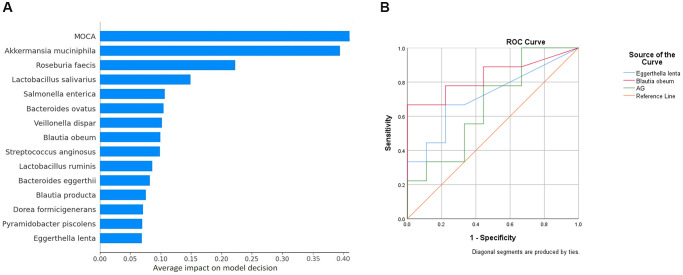
(**A**) Important features contributing to neural network model decision. The X- axis indicates the percentage of contribution. The above features from a pool of 61 features (including species OTU table (*n* = 53) and clinical parameters (*n* = 8)) made up 100 percent of the model decision. (**B**) ROC curve analysis. Abbreviation: AG: Acylated Ghrelin.

A neural network model was created for classifying dementia and healthy controls taking normalized species abundance data and clinical parameters as features. Data were classified into training, validation, and test datasets. The test dataset showed a model accuracy of 100%. The percentage contribution of individual features in model decision using SHAP was visualized (Figure 3A). Out of the clinical parameters, MoCA scores played a crucial role in model decisions, implying reliability; 14 out of 53 species considered for the model contributed to the model decision. The ROC curve showed a distinction for the classification of dementia (Figure 3B), with *Blautia obeum* having an AUC of 0.83,* Eggerthella lenta* with an AUC of 0.71, and AG with an AUC of 0.66. Of the 53 species, 12 were significantly associated with different clinical parameters with R2 >0.55 in the dementia group (Supplementary Figure 2A). In contrast, 13 in the control group (Supplementary Figure 2B) exhibited significant association in a linear regression analysis.

### Ghrelin and specific species correlate with dementia scores

A Mantel correlation test between influential species and clinical parameters showed a significant association between *Fecalibacterium prausnitzii* and MoCA (r² = 0.46, *p* = 0.02) in the dementia group (Supplementary Figure 3A), while *Prevotella copri* with AG/UAG ratio (r² = 0.4, *p* = 0.01), and *Bifidobacterium longum* with MMSE (r² = 0.53, *p* = 0.03) in the control group (Supplementary Figure 3B). Similarly, between differential features (species) in the neural network model and clinical parameters, a significant association was observed between *Veillonella dispar* with socioeconomic status (SES) (r² = 0.58, *p* = 0.03), *Blautia obeum* with MMSE (r² = 0.37, *p* = 0.02), *Pyramidobacter piscolens* with age (r² = 0.47, *p* = 0.004), *Blautia producta* with AG/UAG ratio (r² = 0.51, *p* = 0.01), *Bacteroides eggerthii* with age (r² = 0.41, *p* = 0.03), and AG (r² = 0.47, *p* = 0.01) in the dementia group (Supplementary Figure 4A). In the control group, *Streptococcus anginosus* was associated with MOCA (r² = 0.44, *p* = 0.03), and *Roseburia faecis* was associated with AG (r² = 0.52, *p* = 0.01) (Supplementary Figure 4B), respectively.

### Latent variable 2 significantly delineates dementia

**Figure 4:**
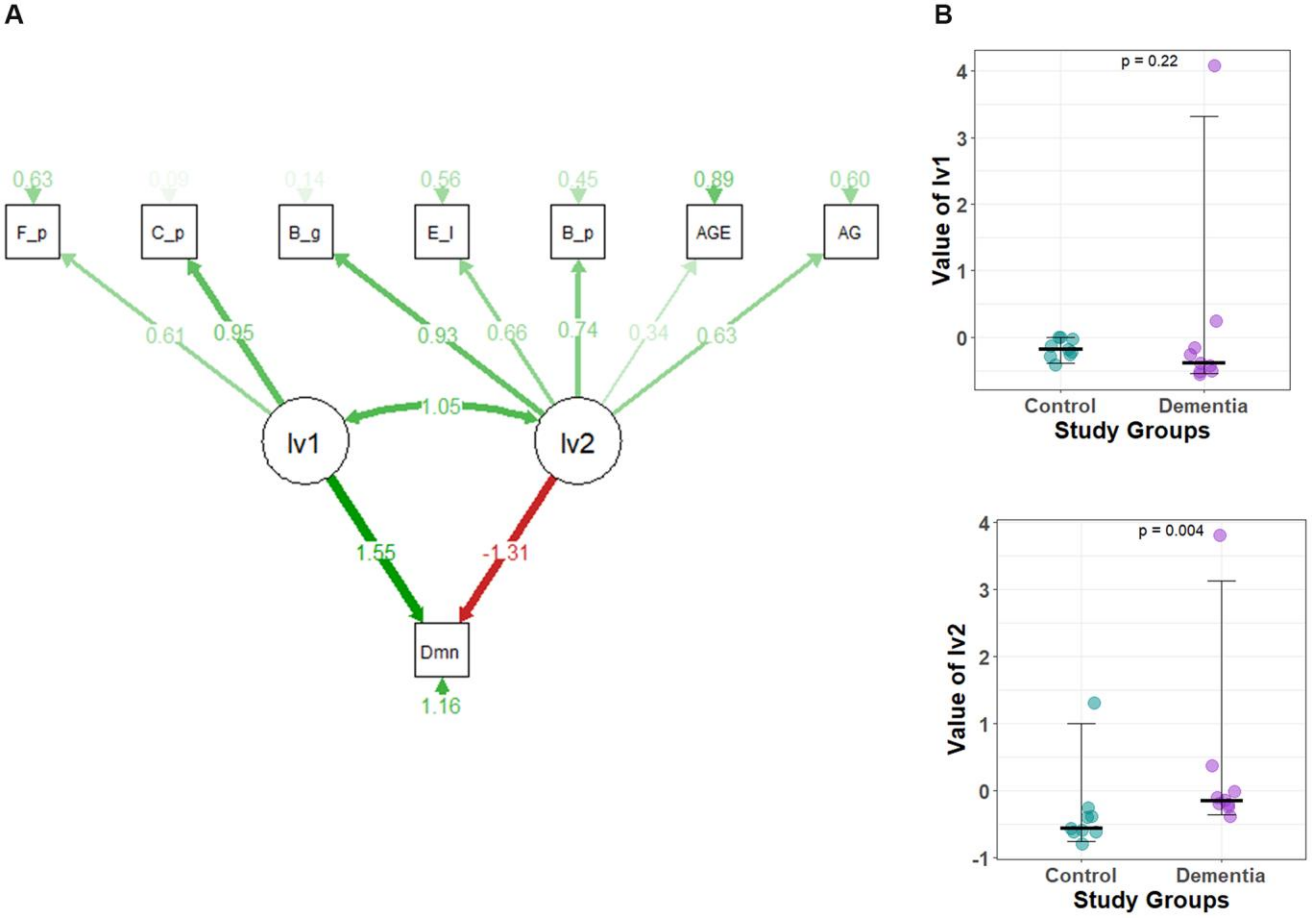
(**A**) The structural equation model (SEM) for dementia. A circle represents latent variables (lv1 and lv2), and a square represents observed variables or indicators. (CFI = 0.797, GFI = 0.697, X^2^/df = 2.083). The darker the lines, the stronger the association. Digits show regression coefficients. F_p, *Faecalibacterium prausnitzii*; C_p, *Clostridium perfingens*; B_g,*Bacteroides eggerthii*; B_p, *Blautia producta*; E_l, *Eggerthella lenta*; AG, Acylated Ghrelin; Dmn, dementia; lv1, latent variable1; lv2, latent variable2; AGE, Age of Study Participants. (**B**) Comparison of the latent variable values between study groups obtained for each parameter of the structural equation model defined in Figure 4A. Latent variable values for (1) lv1 and (2) lv2.

An SEM model delineates the effects of a species group along with age and AG in dementia (Figure 4A). Initially, we eliminated species with no impact and non-significant indications of latent variables. *Prevotella copri* and *Collinsella aerofaciens* were removed from lv1, and *Collinsella stercoris*, *Ruminococcus gnavus*, *Bifidobacterium adolescentis*, *Eubacterium biforme*,* Bifidobacterium longum*, and *Lactobacillus ruminis* were removed from lv2. The final model shows lv1 having a positive effect on dementia (1.55), while lv2 shows a negative impact on dementia (−1.31). The standardized factor loading values from lv1 to each of the indicators of lv1 were as follows: 0.61 for *Fecalibacterium prausnitzii* and 0.95 for *Clostridium perfringens*. Similarly, standardized factor loading values from lv2 to each of the indicators of lv2 were as follows: for *Eggerthella lenta* 0.66, *Bacteroides eggerthii* 0.93, *Blautia producta* 0.74, for age 0.34, and AG 0.63. The lv2 shows a significant difference, while lv1 doesn’t show any differences between the control and dementia groups (Figure 4B).

## DISCUSSION

The significant difference in dementia assessment scores: MMSE, MoCA, GADL, and HADS indicated that the cases and controls clinically differ in their cognition levels. Our findings showed an increased level of AG among dementia patients compared to healthy controls, while UAG levels remained unchanged, replicating earlier study [4]. Hence, the ratio of AG/UAG is significantly higher in dementia cases than in healthy controls, which is contradictory to the previous finding where plasma AG/UAG was found to be decreased among PDD patients [5], implicating normal functioning of the enzyme ghrelin-O-acyltransferase (GOAT), which catalyzes ghrelin acylation in this patient cohort. Increased AG in dementia patients might be a compensatory response to underlying neuronal damage, probably due to decreased neurogenesis or increased neuroinflammation.

Ghrelin is the ‘hunger’ hormone and hence subjected to changes with fasting levels and BMI. Insulin, being released in response to blood sugar is also subject to the same confounding factors that will affect ghrelin. As our mentioned insulin values do not vary significantly between cases and controls, it is unlikely that ghrelin also varies between the groups due to confounding factors. This excludes confounding factors from our analysis and points to the significant difference in ghrelin being associated with cognitive impairment of the patient.

On the other hand, AG is described to have a vital anti-inflammatory action to improve leaky gut evoked by lipopolysaccharides [11, 12]. Since AG was found to be increased in dementia and with AG >1, species richness was found to be decreased, implying underlying leaky gut. Previous studies reported AG as a potential biomarker for neurodegenerative diseases. In our study, the total number of species was significantly higher in patients with AG <1 ng/ml compared to patients with AG >1 ng/ml in the dementia subgroup, another indicator of increased AG with dysbiosis; this implicating AG’s potential role in both ends of the gut-brain axis.

We found that *Prevotella copri* and* Eubacterium biforme* had reduced frequency and abundances in dementia, conforming to previous findings of a decrease in both these microbes in neurodegenerative diseases [13]. Further, our Mantel correlation analysis showed that *Prevotella copri* was significantly associated with AG/UAG in controls. *Prevotella copri* is an SCFA-producing bacteria that helps maintain an intact mucosal barrier at the gut lining*,* contributing to its anti-inflammatory role. *Prevotella copri*, infact alleviates the oxidative stress induced neurological deficits that might be involved with dementia pathogenesis [14]. Our results show an overall incidence of 100% and an abundance of 33% of *Prevotella copri* in all the healthy controls, implying it may be protective against dementia or the predominant microbe of a healthy Indian gut. The beta diversity was markedly different between the dementia and the control group. *Fecalibacterium prausnitzii, Prevotella copri, Eubacterium biforme,* and* Lactobacillus ruminus* were responsible for the differences in beta diversity. *Fecalibacterium prausnitzii*, a contributing species to the differences in beta diversity, correlated with MoCA scores in both Mantel and linear regression correlation analysis, aligning with a previous study [15]. *Fecalibacterium prauznitzii* is implicated in reducing the intestinal permeability by increased expression of tight junction proteins, decreased release of serotonin and tissue cytokines in chronic low-grade inflammations, which is relevant to leaky gut in the background of dementia [16]. Moreover, *Fecalibacterium prausnitzii* was found to have higher abundance and incidence in controls, suggesting it has a neuroprotective property owing to its anti-inflammatory role [17].

The species co-occurrence network showed that specific species interact more by dominating distinct communities in controls and dementia, suggesting an altered community structure. *Prevotella copri*, *Collinsella aerofaciens*, and *Clostridium perfringens* had a higher influence within the control network of which *Prevotella copri* is present in all the control participants and has an enhanced centrality in the control network, complementing the abundance data where it had 100% abundance in the control group.

Our results show that in controls, *Clostridium perfringens* is positively associated with AG, which is known to cause acute gastrointestinal infection with ranging severity of diarrhoea. This bacterium has found its place in regulating the inflammatory genes in the gut-brain axis [18].

The species showing increased centrality in the dementia network are *Bifidobacterium adolescentis, Blautia producta, Eggerthella lenta,* and *Eubacterium biforme. Bifidobacterium adolescentis* has been shown to increase the production of GABA, an inhibitory neurotransmitter [19]. GABAergic dysfunction has been associated with memory loss in Alzheimer’s dementia [20]. Hence, the enhanced centrality of these microbes in dementia may imply a pathogenic role. *Eggerthella lenta* showed an enhanced centrality in the dementia network and significantly contributed to the decision on the neural network model.

Out of the 61 features used in model training,14 bacteria were found to be in the features that contribute to 100% of model decisions. Our study found *Akkermansia mucinphilia* contribution almost equivalent to MoCA where the latter is an established dementia scoring scale.

Further, to identify whether these bacteria could be discriminatory markers between dementia patients and healthy subjects, we further conducted a mantel correlation analysis to test these bacteria’s correlation with clinical parameters. Among the bacteria that showed significant correlation, *Blautia obeum* correlated positively to MMSE in the dementia group. Moreover, it also had an AUC of 0.86 in our ROC curve analysis for classifying dementia. Belonging to the same *Blautia* genera, *Blautia producta* showed an enhanced centrality in the dementia network and was among the species contributing to the neural network model decision. A decrease in *Blautia obeum* has been associated with neurological disorders, and dysbiosis in the *Blautia* genera has been associated with cognitive decline [21, 22]. These studies, along with our results, show that *Blautia obeum* and *Blautia producta* are likely to be associated with the development of dementia.

The coefficients associated with each latent variable (lv1 and lv2) to dementia were of opposite signs, further asserting the validity of our separation into groups based on impact on dementia. lv2 values differed significantly between controls and dementia patients, implying the use of lv2 as a prospective biomarker in diagnosing dementia. Further, *Blautia producta*, *Eggethella lenta,* and *Bacteroides eggerthii* are likely to be closely associated with the pathogenesis of dementia.

This interplay of microbiome and ghrelin is displayed with the significantly different values of lv2 as it utilizes both AG and species indices for its evaluation. The cross-sectional nature of the study limits the scope to understand the casual relationships.

Although a smaller sample size limited the study, overall, it provides evidence regarding the shift in microbial diversity as dementia progresses. Considering the low sample size, it is important to acknowledge that overfitting to the data may be a possible factor contributing to the network’s 100% accuracy in classifying data. The factors contributing to model accuracy still provide important insight into influential species in the dysbiosis seen in dementia and must be studied in future longitudinal studies. By including intestinal permeability markers, neuroinflammatory markers, and advanced brain imaging investigations, we could have improved our understanding of the role of altering gut microbiome and its association with increased AG in the pathogenesis of dementia. This study also provides a basis and potentially useful biomarkers which can be further explored in longitudinal studies to provide insight on the causative, rather than associative nature of the described bacteria.

## METHODS

### Study design and participant description

**Figure 5:**
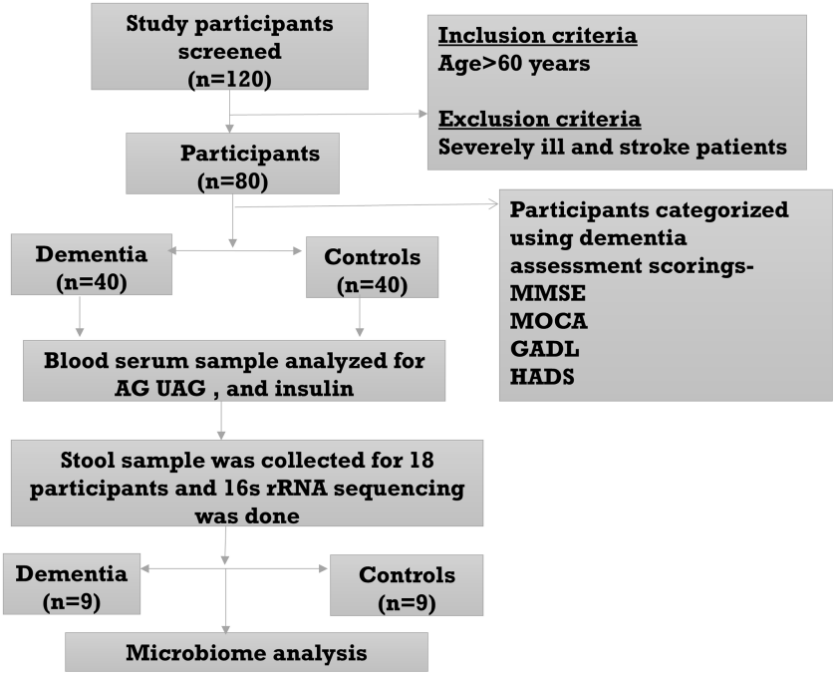
Flow diagram of the study.

This cross-sectional observational study included participants aged ≥60 years. Trauma, stroke, and malignancy-induced dementia subjects were excluded from the study. A total of 40 clinically diagnosed dementia patients and 40 age, gender, and geography-matched controls were recruited from the Neurology OPD, All India Institute of Medical Sciences, Bhubaneswar, Odisha, India. Dementia assessment was done using clinical scores such as MMSE (Mini-Mental State Examination), MoCA (Montreal Cognitive Assessment), GADL (General Activities of Daily Living Scale), and HADS (Hospital Anxiety and Depression Scale). The flow chart of the study design is shown in Figure 5. Demographic parameters like age, sex, education, alcohol and smoking history and clinical parameters like MMSE, MoCA, GADL, HADS, AG, UAG, AG/UAG, AAGR, and insulin were compared in Table 1. n referred to sample size. Ethics approval was obtained from the Institutional Ethics Committee (IEC Ref #: IEC/AIIMS BBSR/PG Thesis/2020-21/107), AIIMS Bhubaneswar. Written informed consent was obtained from all the study participants, and all research was performed following the Declaration of Helsinki.

### Sample size

A sample size of 40 was calculated in each group to get the power of the study to 80% with an effect size of 0.631 based on the mean and pooled SD values of AG from earlier findings [5]. Nine participants from each group were selected for microbiome analysis.

### Serum sample collection

After obtaining informed written consent, a 5 ml venous blood sample was collected from all the study participants after an overnight fast of 8–10 hrs. This sample was centrifuged (Labline brushless centrifuge) for serum separation, and serum was aliquoted and stored at −20°C until further analysis. AG and UAG were estimated per the manufacturer’s instructions using the Human AG ELISA kit and Human UAG ELISA kit, respectively (Bioassay Technology Laboratory PVT Ltd., Zhejiang, China). Insulin was measured using a Human Insulin ELISA kit (Biogenix INC. PVT. Ltd, Lucknow, India) per the manufacturer’s instructions. Student *t*-test was used for pairwise comparison of clinical scores, Ghrelin levels, AG/UAG ratio, and age-adjusted ghrelin ratio (AAGR) between dementia and controls. AAGR = 6+ (log10 (AG/UAG))-(Age/10).

### Fecal sample collection, DNA isolation, and sequencing

Ten grams of fecal samples were collected from study participants, and 0.2 g of stool was used to isolate DNA with a modified DNeasy Powerlyzer PowerSoil kit (Cat No: 12855-100, Qiagen, Qiagen GmbH, Germany). DNA concentration was assessed with a Qubit 4.0 fluorometer (Thermo Fisher, Singapore). Twenty-five nanograms of DNA were then utilized to amplify the 16S rRNA hypervariable V3-V4 region using Illumina MiSeq [23]. All samples that passed the QC threshold (Q20 >95%) were used for further analysis.

### Raw reads processing and microbiome analysis

Raw reads were decontaminated by adapter and barcode removal using TrimGalore v0.6.10 [24]. Low-quality reads were trimmed using Fastp [25]. Taxonomic profiling was performed using Kraken2 [26], aligning it into the GREENGENES v.13.8-99 database. The reads were clustered into OTUs (Operational Taxonomic Units) with a similarity threshold of 97%. For microbiome analysis, we had set a filtration criterion for making the species OTU table where a total count of more than 10 was considered. The relative abundance of species and the relative abundance vs. incidence of the top 15 abundant species was calculated, where incidence is taken as the presence of species in respective groups’ samples when abundance is >1%.

### Diversity analysis

Alpha diversity was assessed for control and dementia groups and subgroups based on AG concentration (AG >1 ng/ml and AG <1 ng/ml). In Alpha indices species richness, shannon diversity, simpson diversity, pielou’s evenness was measured, A Student *t*-test was used to compare alpha indices between the control and dementia groups. At the same time, beta diversity was estimated using the Bray-Curtis dissimilarity index, plotted on PCoA (Principal coordinate analysis) ordination, followed by a PERMANOVA test used for comparison in beta diversity using the Adonis function. Both alpha and beta diversity were estimated using vegan package [27]. SIMPER test was used to identify the species that distinguished groups based on the Bray-Curtis dissimilarity in beta diversity. A natural log-transformed F/B ratio (Firmicutes to Bacteroidetes) was calculated, and a *t*-test was used for statistical comparison between control and dementia. The ggpubr package was used for statistical analyses [28]. Linear regression model was used to find the interaction between species and clinical parameters in the control and dementia groups. All the statistical analysis was performed using R. A *p*-value < 0.05 is considered statistically significant.

### Neural network

A multi-layered perceptron neural network (MLPNN) was used to predict diseased conditions (Dementia) from controls. Normalization of compositional data (scale/centre) was done on all samples with total abundance across all samples greater than 10. Disease condition was classified into binary numbers with controls, and dementia represented as 0 and 1, respectively. A validation and test dataset of controls and diseased patients were separated after shuffling to prevent bias and overfitting. Hyperparameters were optimized using ADAM optimizer. The model was created using TensorFlow on Python [29]. The contribution of features to accuracy on the test dataset was assessed using the SHAP package on Python. Test accuracy and contributing features were calculated [30].

### Co-occurrence network analysis

Networks were created separately for the patients and control group using the MicNet Toolbox [31]. The toolbox uses the SparCC method of creating correlation matrices for compositional data [32]. Iterative SparCC was done with 20 iterations and log transformation of data. Communities of bacteria were created using the Louvain method. Representative high-degree bacteria in both networks were labeled. The degree centralities of each node in patients and controls were normalized. The normalized values of the patients’ group were subtracted from the control group and plotted.

### Mantel test

The Mantel correlation test calculated the potential species in beta diversity interaction with clinical parameters. This test was used to determine the interaction between two distance matrices. Bray-Curtis distance matrix was used for species, while Euclidean distance metric was used for clinical parameters. Differential features (Species) in neural network model decisions have been taken for correlation with clinical parameters using the Mantel Test. Mantel test calculation was performed using the R package linkET [33].

### ROC curve

Differential features (Species) in neural network model decision and ghrelin levels were used to classify dementia at different cut-off values in sensitivity vs. 1-specificity receiver operating characteristic curve (ROC) was generated using SPSS v.25.0 software [34]. The cut-offs were set at >0.65 for clinical data and >0.70 for microbes.

### Structural equation model

We constructed a structural equation model (SEM) using normalized (scaled/centered) species abundance, AG, and age data for dementia and control groups. The SEM was constructed using the CFA function of the lavaan package [35]. The observed variable representing dementia in the structural equation was set as a categorical variable. To construct the SEM, we assumed the presence of two latent variables, one with a positive effect on dementia and the other with a negative impact, explaining the binary categorical variables representing dementia. Based on previous analyses, we categorized species into two groups, one with a positive effect and the other with a negative impact, based on their abundance and influence. Each group was assigned to one latent variable as an indicator. We removed species with no effect and non-significant indicators for the latent variable. The final model was constructed using the remaining species, age, and AG as observed variables. The overall goodness of fit, including the chi-square (χ2) statistic, degrees of freedom (df), whole model *p*-value, the goodness of fit index (GFI), and the comparative fit index (CFI), was calculated. The lavPredict function was used to calculate both latent variable values (lv1 = latent variable 1 and lv2 = latent variable 2) for each sample. The latent variable values were compared between groups using the Wilcoxon rank-sum test with the ggpubr package. The semPlot package was used to visualize the SEM model [36].

## Supplementary Materials

Supplemental File 1
