# Supplementary material for: Exploring the role of acylated ghrelin and gut microbiome in delineating cognitive health in the elderly
Source: Aging (Albany NY). 2025 Feb 7;17(2):393–407. doi: 10.18632/aging.206200 (PMC11892914; doi:10.18632/aging.206200)
Supplement: Supplemental File 1 [file aging-17-206200-s001.pdf]

SUPPLEMENTARY FIGURES

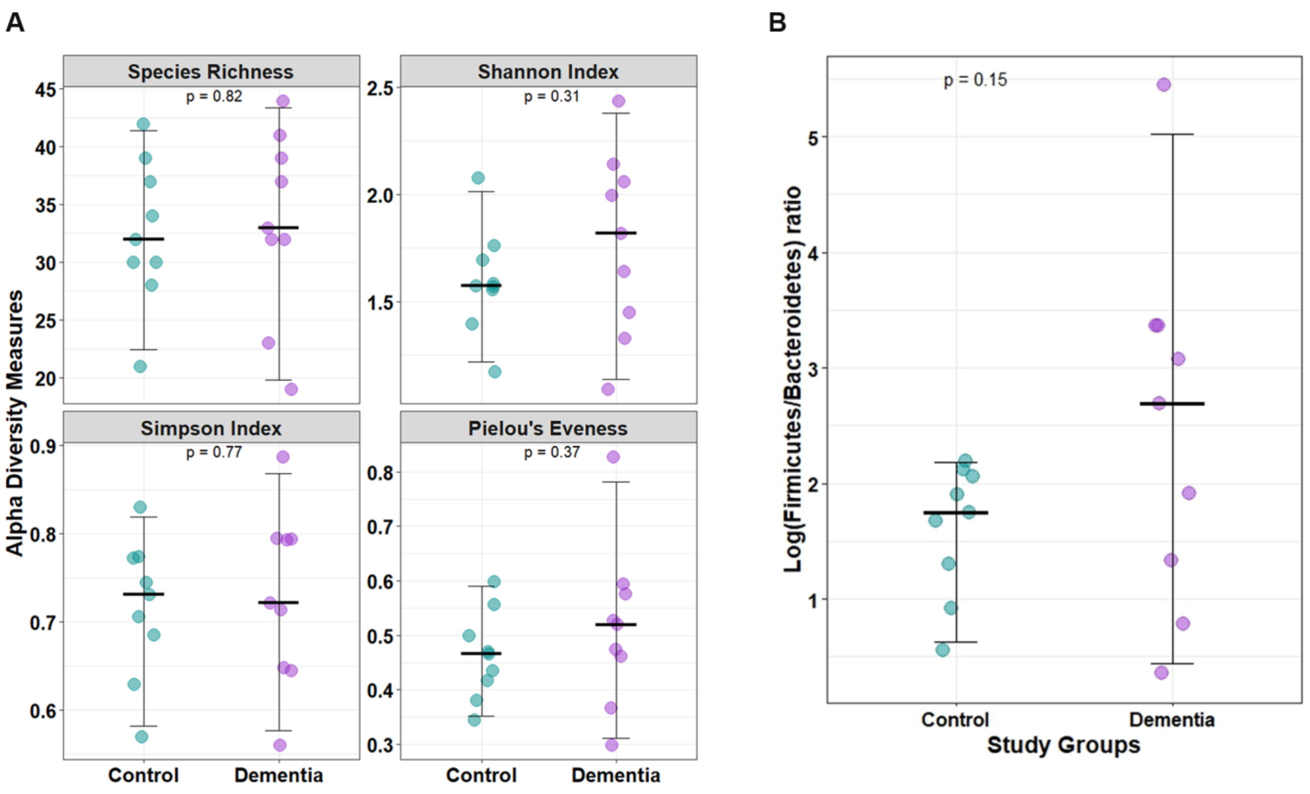

Supplementary Figure 1. (**A**) Alpha diversity comparison between dementia and control group. (**B**) Log transformed Firmicutes/Bacteroidetes ratio comparison between dementia and control group.

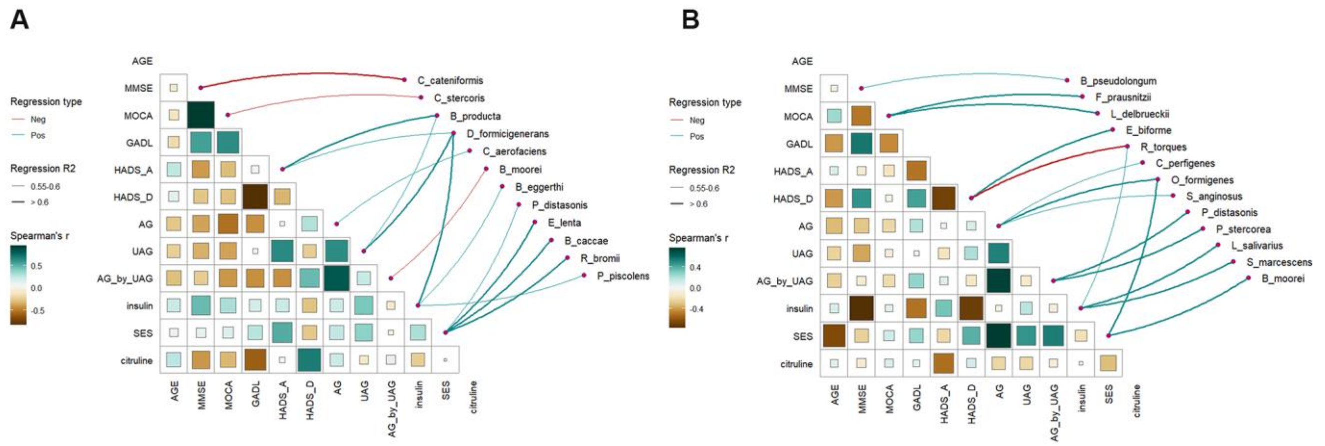

Supplementary Figure 2. Regression between species and clinical parameters. (**A**) Dementia Group, (**B**) Control Group. Red lines show a negative association, and green lines show a positive association. Thicker lines show r2 >0.6.

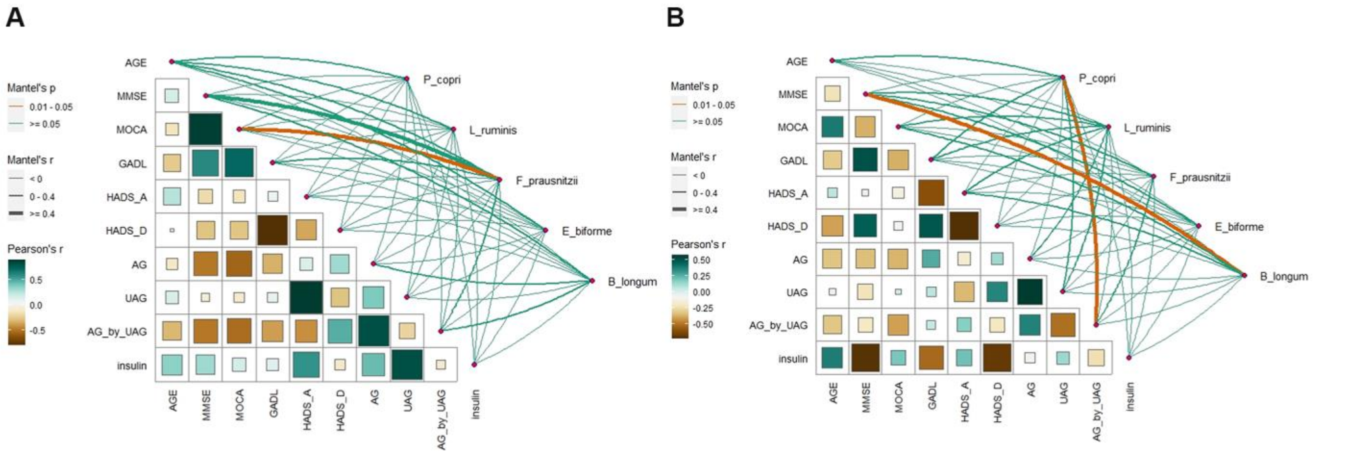

Supplementary Figure 3. Mantel test between influential species in beta diversity and clinical scores and ghrelin levels. (**A**) Dementia Group. (**B**) Control Group. Brown color lines show a significant association ($p < 0.05$).

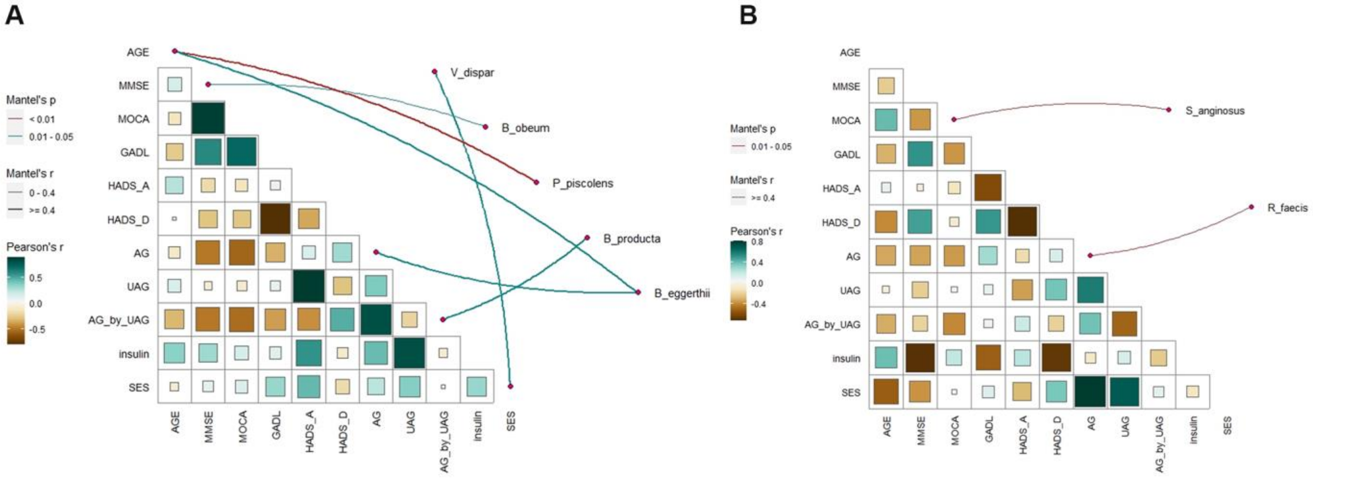

Supplementary Figure 4. Mantel test between important species in neural network and clinical scores and ghrelin levels. (**A**) Dementia group. (**B**) Control Group. Brown color lines show a highly significant association ($p < 0.01$), and dark cyan color lines show a significant association ($p < 0.05$).
